# Effect of early intervention for early-stage psychotic disorders on suicidal behaviours – a systematic review protocol

**DOI:** 10.3389/fpsyt.2024.1359764

**Published:** 2024-02-16

**Authors:** Elkhan Tahmazov, Athéna Blachier, Patrice Nabbe, Morgane Guillou-Landreat, Michel Walter, Christophe Lemey

**Affiliations:** ^1^ Unité de Recherche Clinique Intersectorielle (URCI), Service hospitalo-universitaire de psychiatrie adulte, Centre Hospitalo-Universitaire (CHU) de Brest, Hôpital de Bohars, Bohars, France; ^2^ ER 7479 SPURBO, University of Western Brittany, Brest, France; ^3^ Department of general practice – University of Western Brittany, Brest, France; ^4^ IMT Atlantique, Lab-STICC, Campus de Brest, Technopôle Brest-Iroise, Brest, France

**Keywords:** psychotic disorders in the early stages, prodromic phase, first-episode psychosis, early intervention, suicide, suicide attempt, death by suicide, suicidal ideation

## Abstract

**Background:**

The early stages of psychotic disorders correspond to the early phases of the disease and include the prodromal phase and first-episode psychosis; they constitute a period at high risk of suicidal behaviour. A long duration of untreated psychosis (DUP) is among the risk factors of suicidal behaviour identified in this early period. Many studies have shown the effectiveness of early interventions on the overall prognosis of psychotic disorders in the early stages, and early intervention strategies have been developed and tested worldwide. Several authors reported an improvement in suicidal behaviours; however, all these data have not been systematically analysed yet. The main objective of this systematic review was to collect evidence on the effect on suicidal behaviour of early interventions for patients in the early stages of psychotic disorders.

**Methods:**

We will carry out a systematic review of the literature according to the PRISMA criteria by searching articles in five databases (PubMed, Cochrane, PsycINFO, Scopus, EMBASE), without restriction on the publication date. The selection criteria are: articles (any type; e.g. prospective, retrospective, controlled or uncontrolled, and literature reviews) on early interventions for psychotic disorders in the early stages with data on suicide attempts, death by suicide, suicidal ideation; articles written in English or French. Exclusion criteria are: articles on suicidal behaviours in patients with psychotic disorders in the early stages, but without early intervention, and articles on early-stage psychotic disorders without data on suicidal behaviours.

**Discussion:**

If this review confirms the effectiveness on suicidal behaviours of early interventions for young patients with psychotic disorders, the development/implementation of such intervention programmes should be better promoted.

**Systematic review registration:**

https://www.crd.york.ac.uk/prospero/, identifier CRD42021237833.

## 1 Introduction

Worldwide, more than 700 000 people die due to suicide every year (1), making it a major public health problem with a significant human and economic burden (2). Independently of age, gender and geographical location, psychiatric disorders are the main risk factor of death by suicide and of suicide attempts (3). The psychological autopsy (i.e. the post-mortem analysis of the psychological state) allows determining retrospectively the presence of psychiatric disorders and the type of care received by the patient who completed suicide (4). Data collected with this tool highlighted that the majority of individuals who died by suicide presented a psychiatric history (5).

Psychotic disorders (e.g. schizophrenia) are neurodevelopmental illnesses characterized by altered/loss of contact with reality and impaired functioning, with repercussions on the family and socio-professional life (6). They are diagnosed during adolescence and early adulthood.

It has long been recognized that the onset of overt psychosis is often preceded by a phase of variable duration, characterized by sub-threshold positive and negative symptoms and functional decline (7). In fact, there is a premorbid phase or vulnerability phase during which patients do not present psychotic symptoms, but at are at higher risk of minor psychomotor and cognitive difficulties. Then, a prodromal stage in which patients may present subclinical, unspecific, or transient psychotic symptoms (ultra-high risk mental state), one in which much of the collateral psychosocial damage is known to occur (8), and where we can find moderate neurocognitive changes and functional decline (9). Psychotic transition leads to first-episode psychosis when psychotic symptoms are above the clinical threshold. There is a full threshold disorder with moderate-severe symptoms, neurocognitive deficits and functional decline (8). The interval between the first psychotic symptoms and the first contact with a specialized service is defined as the Duration of Untreated Psychosis (DUP). Recent data indicate that longer DUP is associated with poorer prognosis (6), and that DUP might be an important prognostic factor of the long-term therapeutic response. This can be followed by a chronic phase with unremitting symptoms. In this continuum, the terms of early psychosis covers the prodromal phase and first-episode psychosis.

Suicide is the most important cause of potential years of life lost in young patients with schizophrenia (10). The definitions are as follows (11). Suicidal ideation is defined as thinking about, considering, or planning for suicide. Suicide attempt is defined as a non-fatal self-directed potentially injurious behavior with any intent to die as a result of the behavior. Suicide is defined as a death caused by self-directed injurious behavior with any intent to die as a result of the behavior.

Importantly, in schizophrenia, suicide frequently occurs in the early stages of the disease (12), with an annual incidence 12 times higher than in the general population (13). This trend is particularly noticeable in the first two years after diagnosis (14): the risk of suicide in the first year is increased by 60% compared with the other phases (15). Overall, in patients with early psychosis, the prevalence of suicidal ideation is 40% (16), of suicidal attempts is 8.5-31% (17), and of death by suicide is 0.4%-4.29% (18). Moreover, early psychotic disorders concern mainly a young population, most often between 15 and 35 years of age, and suicide is the second leading cause of death among young people (10 to 34 years of age), after road traffic injuries (19).

Many risk factors of suicidal behaviour have been identified in patients with psychotic disorders in the early stages (20). In their systematic review on suicidal behaviours in patients with first-episode psychosis (18), Coentre et al. listed history of suicide attempt, sexual abuse, substance use disorder (especially cannabis), low overall level of functioning, a negative event, long DUP, older age, more intense positive or negative psychotic symptoms, family history of severe psychiatric disorders, and depressive symptoms. Among these risk factors, the most consistent are history of suicide attempt, depressive symptoms, and long DUP. Some of the risk factors identified by these studies are the same as in the general population, while others are specific to early-stage psychotic disorders, particularly longer DUP. DUP is associated with poorer prognosis (6) and increased risk of suicide. A study on patients with first-episode psychosis found that 70% of them reported suicidal behaviours during the period of untreated psychosis (21). Early interventions reduce the DUP, thus modifying the disease trajectory, and targeting suicidal behaviours (22).

There are various forms of early intervention, including pharmacotherapy, psychotherapy, psycho-social therapies and case-management. Some programs include suicide prevention interventions, such as EPPIC in Australia (23). The duration of these programs can vary, for example it may be 2 or 3 or 5 years. Early intervention takes place in the early stages of psychotic disorders, focusing on the phases of psychotic disorders, and can therefore include minor adolescents or adults, depending on the age of onset of the disorders.

The effectiveness of early intervention on the overall prognosis of psychotic disorders has been demonstrated by many studies, particularly on suicidal behaviours (24–27). Therefore, the main objective of this systematic review is to bring together and assess all evidences to determine whether early interventions in patients with early psychotic disorders decreases the rate of suicide attempts, suicide deaths, and suicidal ideation.

The secondary objectives are to study whether early interventions reduce deaths from all causes; and the impact of the early intervention modalities and duration on suicide attempts, suicide deaths, and suicidal ideation, because there are different program modalities and durations, and it would be interesting to study whether some seem to be more effective than others.

## 2 Methods/design

The protocol of this systematic review of the literature is based on the Preferred Reporting Items for Systematic Reviews and Meta-Analysis Protocols (PRISMA-P) criteria (28).

This study is registered in the international prospective register of literature reviews PROSPERO under the number CRD42021237833.

### 2.1 Eligibility criteria

All articles on early interventions (programs of intervention during early psychosis) in early psychotic disorders (prodromal phase and first-episode psychosis) with data on suicide attempts, deaths by suicide, or suicidal ideation will be included. All articles on early interventions [programs of intervention during early psychosis with different intervention modalities (pharmacotherapy, psychotherapy, case-management, psycho-social therapies, art therapy, adapted physical activity…)].

In psychotic disorders and in young patients, intentionality can be a complex notion, and we can find in this population atypical attempts. In addition to suicide attempts, we also included self-harm, to avoid the bias of underestimating suicide attempts.

We included all-cause deaths in our secondary criteria due to the bias of underestimating suicide deaths, which are not reported as such. We thus want to see if there is an impact on all-cause deaths to see if the results will go in the same direction as those for suicide deaths.

All study types (e.g. prospective, retrospective, controlled or uncontrolled) will be eligible.

Articles on suicidal behaviour in early psychotic disorders but without data on early intervention, and articles on early psychotic disorders without data on suicidal behaviours will be excluded.

**Figure 1:**
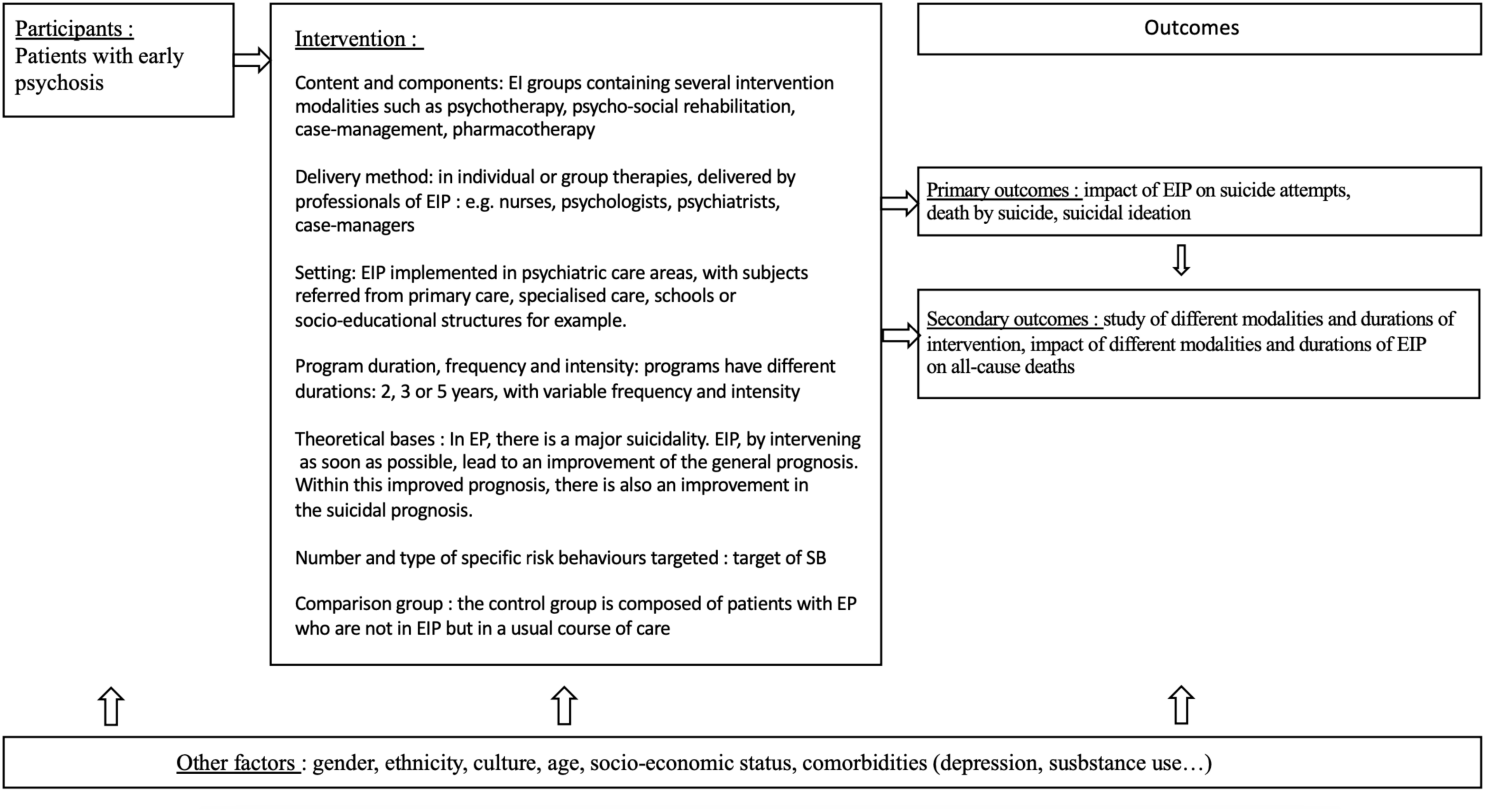
Logic model: early interventions for early-stage psychosis and suicidal behaviours. EI, Early Intervention; EIP, Early Intervention Programs; EP, Early Psychosis; SB, Suicidal Behaviors.

As recommended in the literature (29, 30), **Figure 1** displays the logic model for the proposed review.

### 2.2 Search strategy

To identify relevant articles, the scientific databases PubMed, Cochrane, PsycINFO, Scopus, and EMBASE will be searched without publication date restriction (any article listed from the database creation date), and the languages will be restricted to English and French. The following search query will be used: [(early psychosis) OR (prodromal schizophrenia) OR (at-risk for psychosis) OR (at-risk mental state) OR (high-risk state for psychosis) OR (clinical high risk for psychosis) OR (CHR) OR (ultra-high risk for psychosis) OR (UHR) OR (first-episode psychosis) OR (FEP)] AND (early intervention) AND (suicid*).

The Endnote® bibliographic management software will be used to create a list of the identified articles.

### 2.3 Data extraction and screening

Data will be extracted independently by two researchers (ET and AB). Any discrepancy will be solved by discussion with a third researcher until a consensus will be reached. All retrieved references will be imported into Endnote®. Duplicates will be excluded before data analysis.

A selection will be made after reading the titles and abstracts and based on the exclusion/inclusion criteria. The retained articles will be retrieved as full texts, whereas abstract-only texts (e.g. conference abstracts) will be excluded. The reference list of the included studies will be analysed to obtain other relevant references. The list of retained articles will be completed with bibliographic references of interest from the grey literature.

Data will be extracted by two independent researchers (ET and AB) using a standardized data extraction form for longitudinal studies that will include: publication details (study authors, year of publication), study characteristics (design, country, sample size), intervention characteristics (delivery method, programme duration, content and components, targeted suicidal behaviours), outcomes of interest at all time points (suicide attempts (attempted suicide or self-harm, medicalized or declared)), suicidal ideation (suicidal thoughts, plans, intent), deaths by suicide, all-cause deaths, intervention modalities (pharmacotherapy, psychotherapy, case-management, psycho-social therapies, art therapy, adapted physical activity…), intervention duration (number of years)), measurement tools (validated scales, objective measures), and information on the control group, if applicable.

Data to assess the risk of bias of each study will also be extracted. If necessary, the corresponding author of the included studies will be contacted by email to obtain any required data not presented in the published article.

Data will be entered in the review manager software RevMan version 4.15.0 (31).

### 2.4 Outcomes

The primary outcome measures are the influence of the early interventions for early psychosis on suicide attempts, suicidal ideation, and deaths by suicide. The influence is defined as an increase or decrease in the number of events. Outcome data at all follow-up time points will be extracted and summarized from all selected studies.

The secondary outcomes are the impact of the early intervention modalities and duration on suicide attempts, suicidal ideation, and deaths by suicide, and the impact of the early interventions for early psychosis on all-cause death rate.

### 2.5 Data analysis

All available data will be included in the narrative synthesis. A qualitative synthesis of the data will be conducted: intervention content (targeted suicidal behaviour), delivery method, intervention duration, and sample characteristics. All the available data will be classified and exposed. No quantitative, statistical or meta-analysis studies are planned. Some of the items identified are currently being studied.

The data evidence quality will be assessed with the Grading of Recommendations Assessment, Development and Evaluation (GRADE) system (32).

### 2.6 Risk of bias

Two reviewers (ET and AB) will independently assess the risk of bias of the included studies. Discrepancies will be solved by discussion with the other authors. Biases will be discussed qualitatively, and will be explored in the GRADE tool (32). The GRADE method has been endorsed by many well-known organizations around the world (33).

## 3 Discussion

As suicidal events are rare from an epidemiological point of view, studies must include a large number of individuals to have enough statistical power to show an effect of the intervention. Therefore, prospective studies on this question may be difficult to set up and the included studies may mainly have a retrospective design. This might result in a lower level of evidence and in selection biases inherent to retrospective cohorts, such as the creation of control groups that are less comparable. In studies without a control group, where suicidal behaviours are monitored only in the group participating in the early intervention, results should be viewed with caution. Indeed, suicidal risk could decrease over time as the natural course of disease, and not as a consequence of the early intervention. This underscores the importance of establishing control groups.

Moreover, it might be difficult to find studies on the prodromal phase. Indeed, as the identification of individuals with subclinical symptomatology is complex, studies are more often focused on patients with first-episode psychosis. This might make difficult to generalize our conclusions to all early stages of psychotic disorders.

Another expected selection bias is the non-differentiation between affective and non-affective psychoses. In the context of diagnostic instability, a first-episode psychosis can lead to the occurrence of a schizophrenic disorder, but also to a mood disorder. This non-differentiation could be problematic and affect the homogeneity of the results. Indeed, the integration of affective psychoses might lead to a symptomatology characterized mostly by mood problems, and therefore to more marked suicidal behaviours.

As only five databases (PubMed, Cochrane, PsycINFO, Scopus, EMBASE) will be used for the literature search, relevant articles might be missed. Even if the analysis of the included articles references will lower that risk the publication bias must be taken into account. Studies on the subject may have been carried out but not published by the authors because of the absence of positive results. This could lead to an overestimation of the effect of early interventions for early psychosis on suicidal behaviour.

As early intervention programmes are developed and implemented in some countries more than others, a geographic heterogeneity in the studies should be expected.

The relationship between early intervention and outcome measures may be subject to confounding bias, due to the difficulty of performing blinded studies, and the lack of randomization in some studies.

Different measurement biases also might be expected for deaths by suicide, particularly their underestimation (34), and also different procedures and practices in the investigation, recording, and coding of these deaths among countries (35).

Moreover, variations in the measure of suicidal ideation can be explained by its staging or not, as well as by the different psychometric scales used (e.g. Brief Psychiatric Rating Scale, Beck Depression Inventory, Suicidal Ideation Questionnaire).

For all criteria, different intervention durations and modalities might affect suicidal behaviours differently. In addition, the presence of composite criteria would complicate the study comparison.

This systematic review of the literature on the impact of early interventions in early-stage psychotic disorders on suicidal behaviour will be the first to our knowledge to address this subject.

A systematic review on the effectiveness of suicide prevention strategies in early psychosis could highlight their positive effects on suicidal behaviours, and thus on mortality, and serve as an additional argument to promote the development/implementation of early intervention programmes.

## Ethics statement

Ethical approval was not required for the studies involving humans because this study is not interventional, but as it is a review, we did not submit it for ethics approval. The studies were conducted in accordance with the local legislation and institutional requirements. Written informed consent was not obtained from the minor(s)’ legal guardian/next of kin, for the publication of any potentially identifiable images or data included in this article.

## Author contributions

ET: Conceptualization, Data curation, Formal analysis, Investigation, Methodology, Project administration, Resources, Software, Supervision, Validation, Visualization, Writing – original draft, Writing – review & editing. AB: Conceptualization, Data curation, Formal analysis, Investigation, Methodology, Project administration, Resources, Software, Supervision, Validation, Visualization, Writing – original draft, Writing – review & editing. PN: Conceptualization, Data curation, Formal analysis, Investigation, Methodology, Project administration, Resources, Software, Supervision, Validation, Visualization, Writing – review & editing. MG-L: Conceptualization, Data curation, Formal analysis, Investigation, Methodology, Project administration, Resources, Software, Supervision, Validation, Visualization, Writing – review & editing. MW: Conceptualization, Data curation, Formal analysis, Investigation, Methodology, Project administration, Resources, Software, Supervision, Validation, Visualization, Writing – review & editing. CL: Conceptualization, Data curation, Formal analysis, Investigation, Methodology, Project administration, Resources, Software, Supervision, Validation, Visualization, Writing – review & editing.
